# Eye tracking based dyslexia detection using a holistic approach

**DOI:** 10.1038/s41598-021-95275-1

**Published:** 2021-08-03

**Authors:** Boris Nerušil, Jaroslav Polec, Juraj Škunda, Juraj Kačur

**Affiliations:** Institute of Multimedia ICT, Slovak University of Technology in Bratislava, Bratislava, Slovakia

**Keywords:** Electrical and electronic engineering, Computer science, Scientific data, Preclinical research

## Abstract

A new detection method for cognitive impairments is presented utilizing an eye tracking signals in a text reading test. This research enhances published articles that extract combination of various features. It does so by processing entire eye-tracking records either in time or frequency whereas applying only basic signal pre-processing. Such signals were classified as a whole by Convolutional Neural Networks (CNN) that hierarchically extract substantial features scatter either in time or frequency and nonlinearly binds them using machine learning to minimize a detection error. In the experiments we used a 100 fold cross validation and a dataset containing signals of 185 subjects (88 subjects with low risk and 97 subjects with high risk of dyslexia). In a series of experiments it was found that magnitude spectrum based representation of time interpolated eye-tracking signals recorded the best results, i.e. an average accuracy of 96.6% was reached in comparison to 95.6% that is the best published result on the same database. These findings suggest that a holistic approach involving small but complex enough CNNs applied to properly pre-process and expressed signals provides even better results than a combination of meticulously selected well-known features.

## Introduction

The International Dyslexic Association and the US National Institute of child health and human development have defined dyslexia as a specific learning disability that is neurobiological in origin^1^. Worldwide, it is estimated that about 10% of the population suffers from dyslexia. Dyslexics face issues while processing words from written language to speech and in a word decoding. The result is an inability to read or a very cumbersome reading. Typical manifestations of dyslexia include problems in distinguishing shapes of letters, not being able to separate sounds that are similar, such as *b, d, p, q, m, n,* etc. The rotation of letters can be considered as an effect that causes these difficulties. Moreover, for dyslexics it is hard to recognize letters that are in wrong positions^2^. Other manifestations of dyslexia include omitting letters and syllables within words and misunderstanding the content of the quoted text^3,4^. As a consequence, it has been observed that the eye movements of dyslexics are different from the eye movements of non-dyslexics. Typical patterns for dyslexic eye movements are more frequent and longer fixations, shorter saccades and more regressions. In^5^ fixations are specified as periods when gaze remain at the same location for 200–300 ms and saccades as rapid and abrupt eye movements that shifts the point of fixation. Eye movements from right to left are called regressions whereas those from left to right are marked as progressive, and both can be considered as types of saccadic movements.

It is assumed that the increased word processing requirements lead to the longer processing time and changes in fixation patterns^6^. These observations can be considered as basic assumptions for the research branch focusing on eye movements. Several studies compared the eye movements of dyslexics and normal readers and many types of eye movement have been shown to have diagnostic potential for subjects with dyslexia, e.g. for dyslectics it is typical to make more regressions than healthy ones^7,8^.

**Figure 1:**
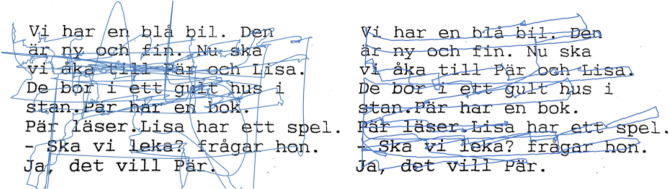
Eye movements of randomly selected subjects from the high risk (left) and low risk (right) dyslexia groups during a text reading test (movements outside the paper are not shown because of spatial reasons).

Children with dyslexia require special learning methods to overcome reading difficulties. To diagnose dyslexia, it is necessary to perform assessments either in written or oral form^9^. By tracking eye movements during reading it is possible to create a path of visual attention^10,11^ as shown in Fig. 1. Cognitive defects in subjects can be detected by detailed investigation of the visual path. Various types of cognitive disorders as autism, schizophrenia, dementia can be identified by using eye tracking technology^10,12^. Moreover, there are many articles on modelling human visual attention, e.g.^13^ as well. Currently, there are several research projects detecting cognitive disorders by evaluating visual attention of subjects either while viewing pictures or regarding texts. Such scenarios combined with an eye-tracking form a base for the detection of cognitive disorders^12,14–17^. In some cases regions of interest in selected pictures are analysed, e.g. cards of the Rorschach test (ROR) for the detection of schizophrenia^14^, whilst in the detection of other cognitive disorders (autism, dementia, or dyslexia) the text reading test is of the main focus^12,18–20^. This article is especially dedicated to dyslexia detection where the eye movements of dyslectic are assumed to differ from healthy individuals^16,20^.

The article is organized as follows. First, an overview of the related work is provided focusing on the detection of dyslexia. Then a detailed description of the proposed approach is outlined together with the supporting assumptions. In paragraph 4, the dataset, training and testing conditions are presented. Next the designed experiments and results are shown. The article is concluded by summarizing the achieved results in comparison to the reference methods, and concluding remarks.

## Related work

Several research articles have investigated the association between abnormal eye movements and dyslexic disorders during a reading activity. Most of them evaluated their methods on a dataset presented in^16^ comprising 97 subjects of high risk (HR) and 88 subjects of low risk (LR) of dyslexia, i.e. in total there are 185 recordings.

In the original research^16^ fixations were defined as periods when eyes remain steady for at least 50 ms, and saccades where eye movements cross a certain distance threshold. Fixations were used to define set of derived features as duration of an event, distance spanning event, average eye position during an event, standard deviation of average position, maximum range between any two positions, and accumulated distance over all subsequent positions. In total the authors generated 168 features capturing different quantitative eye movement properties during reading that can be categorized as durations, amplitudes, directions, stability and symmetry measures. Such set of distinctive features was reduced down to 48 features by using recursive feature elimination (RFE). The classification was based on support vector machines (SVM) reaching an accuracy of 95.6% and together with a feature elimination it forms the SVM-RFE method^16^. In^21^, the authors used the Particle Swarm Optimization (PSO) and Hybrid Kernel SVM classifier in their approach to detect dyslexia. Statistical methods were used to extract features from eye movements such as fixations and saccades. These features were analysed-processed by Principal component analysis (PCA) and then classified. They reported an average detection accuracy of 95%. In^22^ the authors tested different algorithms such as Random Forest Classifier (RF), SVM or k-nearest neighbours (k-NN) with different features derived from the eye fixations that were further reduced by RFE. In such settings the k-NN classifier recorded the best accuracy of 95%. The authors in^5^ used statistics of fixations and saccades and in addition to that they deployed a variance threshold identification (I-DT) and velocity threshold identification (I-VT) algorithms to derive extra features. Then the Hybrid Kernel SVM-PSO achieved the best result of 95.6%, using a feature set containing the average number of fixations and saccadic movements, and the average duration of fixations and saccadic movements. In^15^ the authors used a dataset having 48 subjects with the diagnosed dyslexia and 97 subjects with no dyslexia. The extracted features they used include, e.g. the number and duration of fixations, the number and duration of returns, the age of the subject or the font of the text. The data were classified using a SVM binary classifier recording an accuracy of 80.18%.

Other important group of publications^23–25^ focusing on the diagnosis of dyslexic disorders is based on analysing brain images either in form of Functional Magnetic Resonance Imaging (fMRI) or MRI. The reported accuracies range from 80 to 92.5% depending on the used dataset, processing and classification methods.

Another interesting approach published in^26^ is based on analysing handwriting images of children aiming to identify the symptoms of dyslexia. The neural network (NN) based classification reached a 73.33% accuracy.

## Proposed approach

Our method for the detection of dyslexia is based on analysing eye movements during a text reading activity. Unlike similar methods, we decided to apply a different approach than representing the eye movement signal by many specific features evaluated at the global level. Instead, the eye movements that are represented as sequences of x–y coordinates are modelled and classified as a whole either in time or frequency, i.e. without an explicit feature extraction stage. This is to test if a proper model can both extract “hidden” and possibly more representative features and classify them at the same time using machine learning. It is important even though many designed features show a good performance, however there is still no prove they are optimal for the detection of dyslexia.

Our method as the majority of others consists of two main parts. Nevertheless, due to a holistic approach they are not that strictly separated, i.e. signal processing/feature extraction and a classification stage that are realized by a CNN, which acts as a feature extraction method and a classification tool as well. In the following both will be outlined.

### Signal pre-processing and signal representations

**Figure 2:**
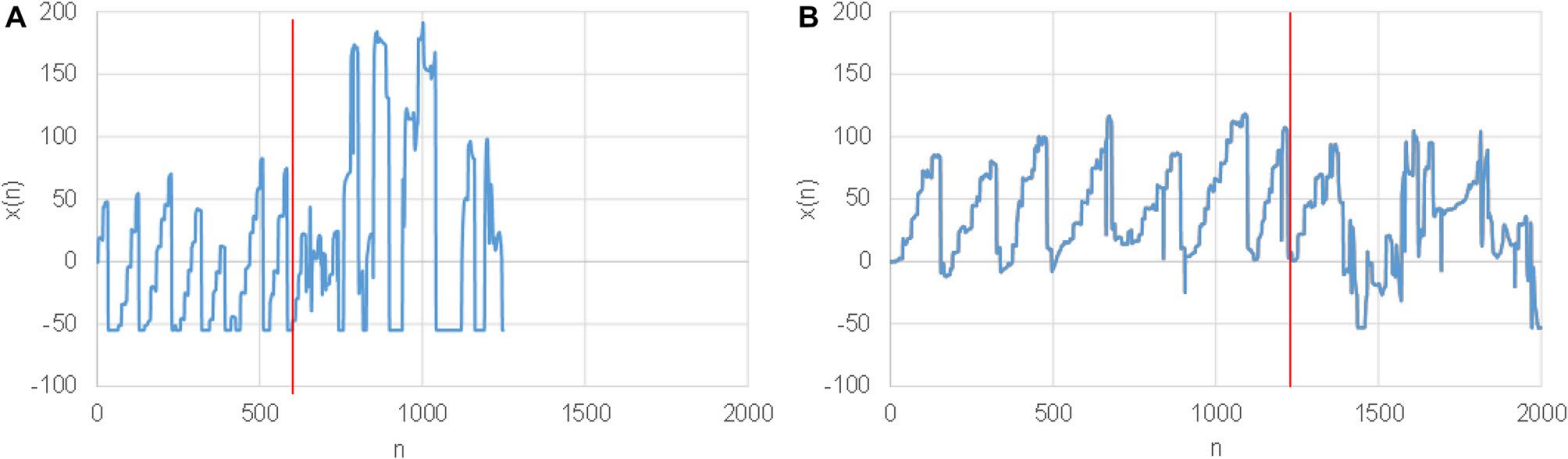
The eye movements expressed in x axis for a typical—LR subject (**A**) and HR subject (**B**). The end of reading part is marked with a red line.

The proposed method was derived following natural and well accepted assumptions as stated in the following:

1. Even though the total text reading time is significantly greater for children with high risk of dyslexia (HR) than for children with low risk (LR), it is not true in all cases.
2. The time course of x-coordinate in LR children has a saw-tooth like shape (Fig. 2A), which ideally has as many maxima (teeth) as there are lines of the read text and each tooth has almost a linear course.
3. The time course of x-coordinate in HR children has also a saw-tooth like shape, however sometimes a sudden backward movement is observed, (Fig. 2B). This naturally deforms the “ideal” saw-tooth as observed in LR subjects.
4. If all LR children read the text as fast as HR children, the spectrum of the saw-tooth like signal according to assumption 2 should be shorter for LR subjects than the spectrum observed for HR subjects according to assumption 3 (sudden returns result in more complex frequency components, i.e. less decaying spectrum).

With regards to assumptions 1–4, the proposed method reflects all of them. To see how relevant assumption 1 is, two options were tested. First one uses signals stripped off the time information by interpolating all recordings to the maximal sample length. We chose a suitable interpolation method based on a discrete orthogonal transformation. The second approach applies a zero-padding of shorter signals to the length of the longest one (the same-length condition was also required by CNN that assumes a fixed length input), where the time information is implicitly present in the form of a number of padded zeros.

To reflect assumption 4 (indirectly 2 and 3) the standard Discrete Fourier transform (DFT) was used to calculate a spectrum of examined signals. More precisely the magnitude spectrum (only its first half) should sufficiently describe the saw-tooth nature of the signal as well as the disproportion in the shape of typical “teeth” for LR and HR children. The additional benefit of the magnitude spectrum is that it does not depend on which line (position) a HR child encountered a reading problem and had to shift his or her gaze to the beginning of a given line (such information is present in a phase spectrum), as we believe these sudden shifts (returns) happen randomly (line independently). Therefore, by removing the positional information we have dramatically reduced all possible combinations of those sudden returns and thus made the CNN training process much easier, i.e. not having to learn all positional combinations that occur randomly. To confirm the frequency based assumptions, signals in the time domain either interpolated (no reading time information) or zero padded (reading time implicitly present) were directly used as CNN inputs too.

Unlike other approaches, here the signal is pre-processed only to meet the basic assumptions as stated before, whereas the genuine feature extraction and classification parts are implicitly realized inside a proper classifier (CNN) that process entire signals (holistic approach).

Eye movements expressed as x–y coordinates for each eye separately i.e. (Rx, Ry) right eye, and (Lx, Ly) left one were averaged over both eyes as there is no evidence that dyslectic patients would have different scanning patterns for left and right eyes. Moreover, averaging of x–y trajectories over left and right eyes produces less redundant and more robust training–testing samples that are easier to process and classify.

Subsequently, all signals were cut in length so that only active reading parts were present. Furthermore, it was observed that after the reading was accomplished (reaching end of the last line) the subjects regardless of LR or HR groups paid attention to different parts of the text exhibiting different eye-movement patterns. It was found that non-reading parts represent approximately half of the recording time, and so this could affect the detection process that is based only on text reading. In Fig. 2A,B entire signals (x-coordinate) with the marked end of reading are shown for a typical LR and HR subjects.

In the subsequent data processing, we have derived and tested following signals that are in line with the outlined assumptions:

- Time domain zero padded signal
- Spectrum of a time domain zero padded signal
- Time domain interpolated signal
- Spectrum of time domain interpolated signal

**Figure 3:**
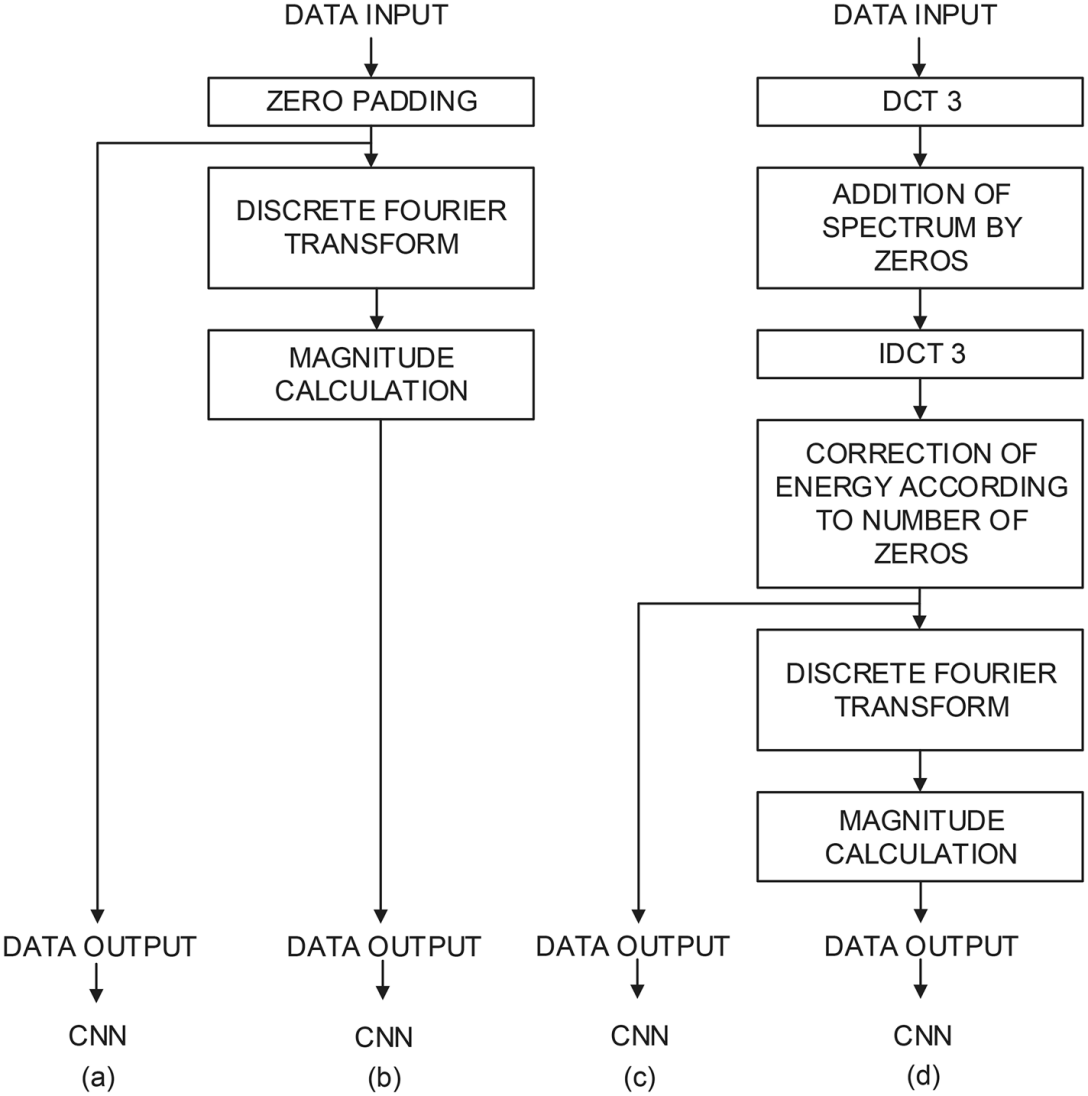
A block scheme of the designed and tested data processing methods; (**a**) time domain zero padded signal, (**b**) spectrum of time domain zero padded signal, (**c**) time domain interpolated signal, (**d**) spectrum of time domain interpolated signal.

Bock schemes illustrating the suggested signal pre-processing methods, e.g. processed signals entering the CNN classifier are shown in Fig. 3.

**Figure 4:**
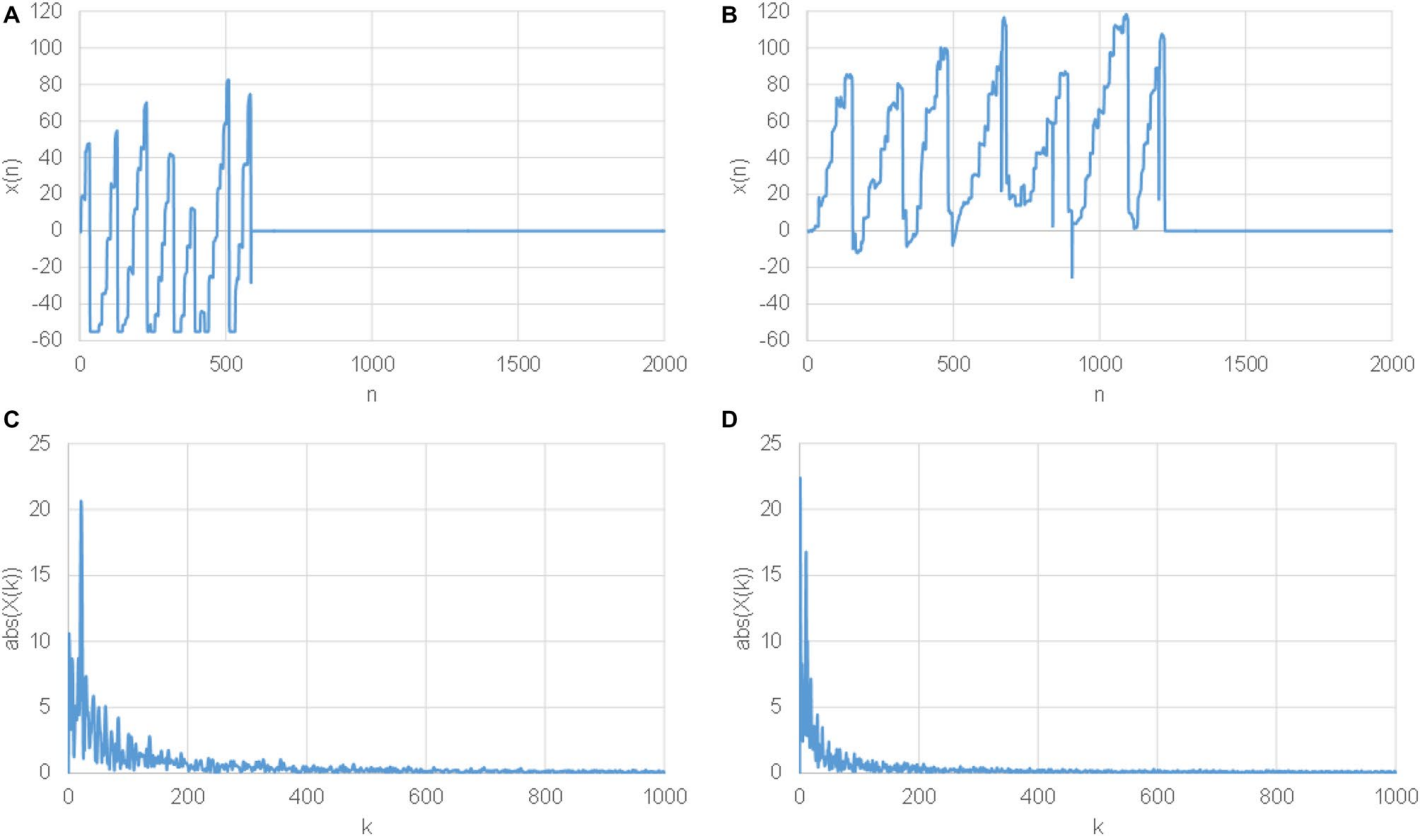
Time domain zero padded eye movement signal (x axis) of a typical LR subject (**A**), and HR subject (**B**). Magnitude spectrum of time domain zero padded signal (x axis) of a typical LR subject (**C**), and HR subject (**D**).

In methods (a) and (b), signals were padded by zeros to get the same length as of the slowest reader. Such signals are shown in Fig. 4A,B, for LR and HR subjects, respectively. In method (b), additionally, the DFT was applied and a magnitude spectrum was calculated, as shown in Fig. 4C,D for LR and HR subjects.

**Figure 5:**
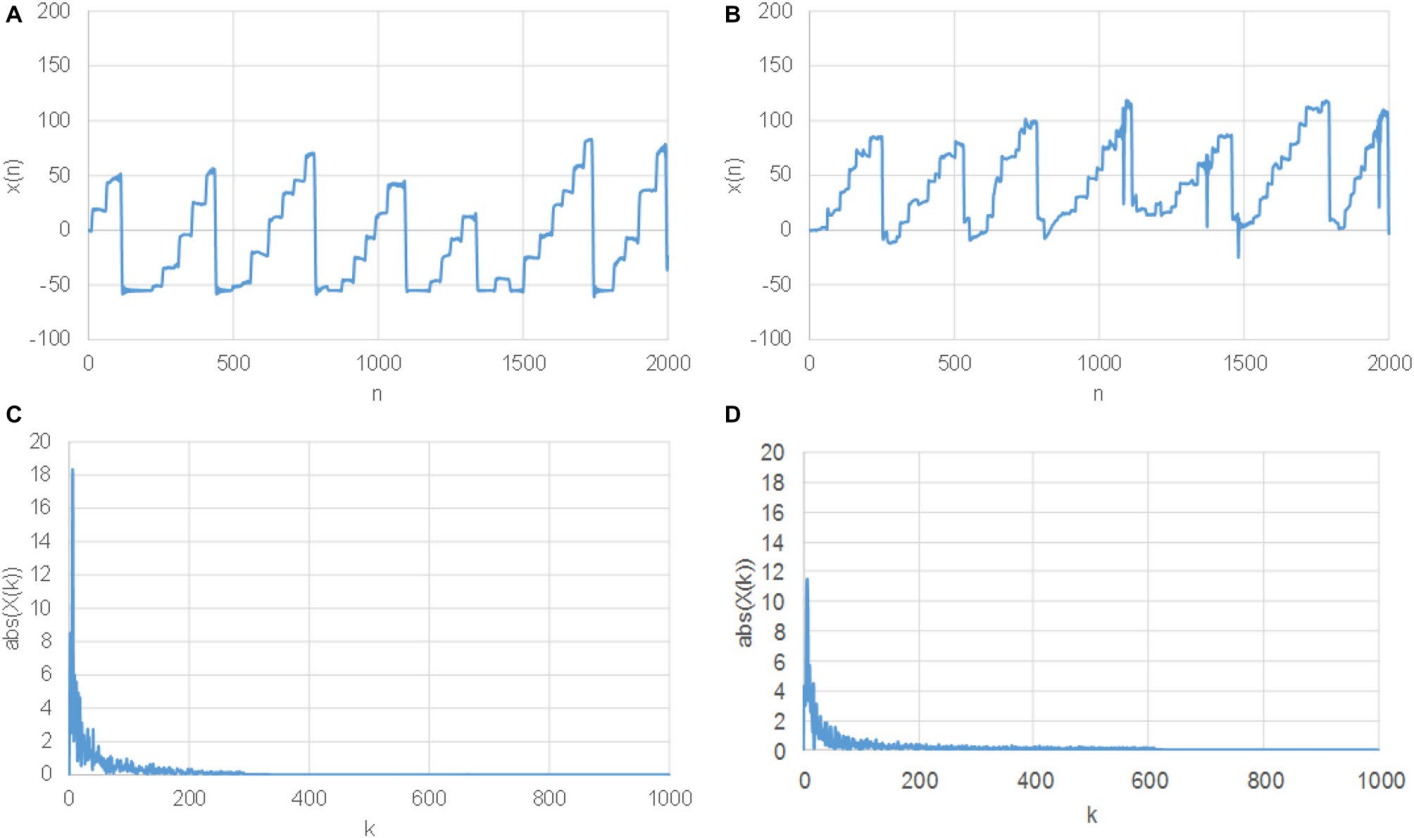
Time domain interpolated signal (x-axis) for a typical LR subject (**A**), and HR subject (**B**). Magnitude spectrum of time domain interpolated signal (x-axis) for a typical LR subject (**C**), and HR subject (**D**).

In methods (c) and (d) a signal interpolation in time is applied to get fixed length signals entering a CNN classifier and to remove the reading time. All signals were interpolated—extended to the length of the slowest reading subject. They were interpolated by harmonic functions preferred in image processing; in our case we used base functions of the Discrete Cosine Transform 3 (DCT3) to get signals of the fixed length N as in Eq. (1):

1 $$ DCT3_{{U_{k,n} }} = \sqrt{\frac{2}{N}}  c_{n} \;\cos \left( {\frac{\pi ((2k + 1)n)}{{2N}}} \right)_{{ k = \, 0,{1}, \ldots ,N - 1, \, n = \, 0,{1}, \ldots ,N - 1,}} $$

where k and n represent spectrum and time indexes, respectively. Next, the ratios between the original and the modified lengths were calculated for each subject. Such interpolated signals were multiplied by the ratios to correct the overall energy; x-axis interpolated signals are shown in Fig. 5A,B for LR and HR subjects. The same signals represented in the frequency domain, i.e. by their magnitude spectra are shown in Fig. 5C,D.

Except the reading time, horizontal signals (x-axes) differ between LR and HR subjects too, as can be seen in Fig. 5A,B. Whilst x axes in case of LR subjects closely resemble a saw-tooth shape, such pretty deterministic structure is disrupted by unpredictable abrupt backward movements and equally sudden returns to the original position. This discriminative pattern was rather dominant (carries significant information) thus the vertical axis (y) was not included in the following processing and classification. By doing so we reduced the amount of processed data by factor 2, the complexity of CNN (kernel dimension), and thus simplified the training process as well. Moreover, experiments involving also y axis were performed, but no improvements were observed.

Finally, it should be noted that a magnitude spectrum representation of signals reduced the amount of process data by factor of 2 (as spectrum is symmetric only 1st half is needed), and that the spectral unidirectional component does not carry any obvious information (just the centre of text in the horizontal direction) and so it was excluded from the final vector entering the CNN.

### Convolutional neural network—suitable classification model

As we decided to process and classify entire signals which contain both the typical patterns for a text reading task and specific ones caused by the reading errors that are randomly scattered in the time, the natural choice was a CNN network^27^. The search for scattered patterns can be performed both in time and frequency domains and thus a CNN structure acts as unifying framework for all our intended experiments. CNNs are currently the best performing NN structures in image and video recognition, image classification, medical image analysis and language processing^15,28,29^. It is due to their structures that allow to extract relevant features located at different positions (shift invariant), more filters (kernels) placed in parallel planes provide a resolution invariance, and proper concatenation of more convolutional layers enables a hierarchical non-linear feature extraction that is vital for very complex features. Thus a CNN usually requires relatively small signal pre-processing compared to other image classification algorithms as it learns the feature extraction model by means of machine learning minimizing a classification error. It is unlike traditional algorithms that use theoretical (usually limited) knowledge to extract particular features prior to the classification stage. This independence from prior knowledge and human errors in the design is a major advantage.

## Training and testing setup

In our dyslexia detection experiments we used database that was presented in^16^ as it meets all the necessary requirements, and to have the same data as most of the other published methods that is vital for a correct comparison. It contains 185 recordings each for a single tested subject. Subjects with cognitive disorder were in the HR group—reading disorder was identified in 97 subjects (76 male and 21 female subjects). In the LR group 88 subjects were presented (69 male and 19 female subjects). The age of subjects ranged between 9 and 10 years, and none of them suffered mental retardation. The text that was read consisted of 10 sentences divided into 8 lines with an average length of 4.6 words. The eye movements were recorded by Goggle-based Obe-2 TM system in the horizontal and vertical directions (x, y axes) for both eyes at the sampling frequency of 100 Hz. For the purpose of our research and taking into consideration the outlined assumptions only horizontal (x-axis) eye movements were averaged over both eyes and used for further processing.

In our experiments, we used Matlab both for the signal pre-processing and the classification that was realized by a CNN. To reduce the effect of improperly selected classification models i.e. CNN complexity, we selected CNNs with different number of convolutional layers, namely 2, 3 and 4. The exact structures and names of the tested CNNs are:

- CCN2—2 layers, kernel sizes [1 × 3, 1 × 3], Relu activation function, number of kernels [8, 16], max pooling [1 × 2] with a stride [1 × 2]
- CNN3—3 layers, kernel sizes [1 × 3, 1 × 3, 1 × 3], Relu activation function, number of kernels [8, 16, 32], max pooling [1 × 2] with a stride [1 × 2]
- CNN4—4 layers, kernel sizes [1 × 3, 1 × 3, 1 × 3, 1 × 3], Relu activation function, number of kernels [8, 16, 32, 64], max pooling [1 × 2] with a stride [1 × 2]

The output of each convolutional layer was batch normalization. At the end a 2 layer fully connected network with a softmax output was appended. In the training Stochastic Gradient Descent algorithm (initial learning speed of 0.01) with a momentum was used to minimize a crossentrophy loss function.

**Figure 6:**
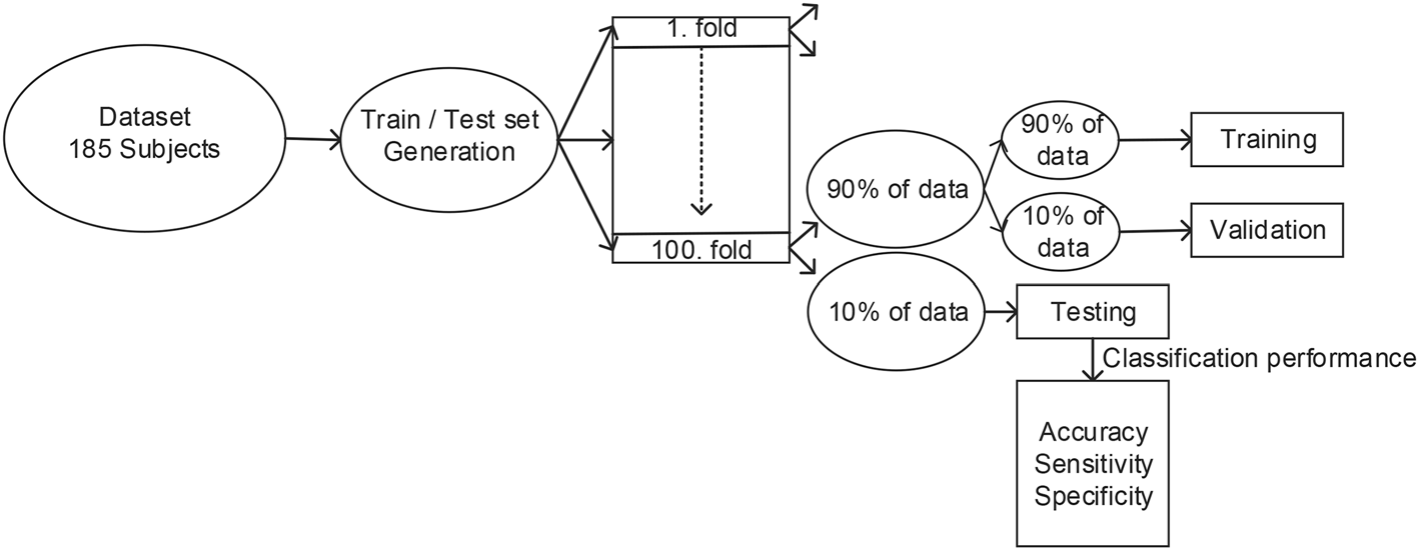
A block scheme of the training—testing process.

Out of 185 subjects we generated 100 different test folds each containing 16 subjects (8 HR, 8 LR). Such scenario mimicked the training conditions as stated in^16^ and provides more accurate and robust results as well. It was ensured that in the worst case there was at most a 50% intersection between any folds (sets). The remaining data were divided as 90% for training and 10% for validation. The training, testing and validation process is shown in Fig. 6.

## Results

Observing the trimmed data and considering assumption 1, we first tested a very simple classification method that is purely based on a histogram of reading times; as reading times of LR subjects are in the majority of cases shorter than in HR group. If only such simple parameter is used it is possible to find a single threshold providing very high accuracy of 95.67%.

**Figure 7:**
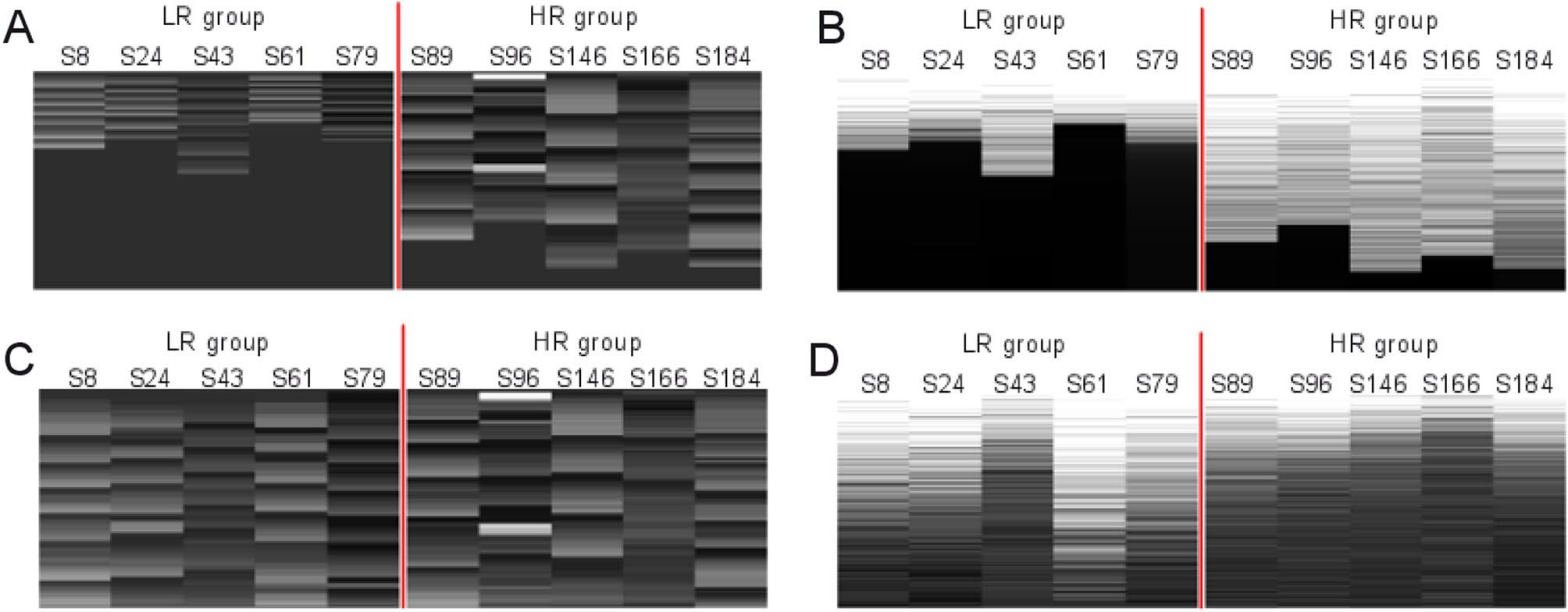
(**A**) Time domain zero padded signals for 5 LR and 5 HR subjects, (**B**) Magnitude spectra of time domain zero padded signals for 5 LR and 5 HR subjects, (**C**) Time domain interpolated and energy normalized signals for 5 LR and 5 HR subjects, (**D**) Magnitude spectra of time domain interpolated and energy normalized signals for 5 LR and 5 HR subjects. Due to the visualization signals/spectra were quantized into the grayscale range < 0–255 > , i.e. 0 is black and 255 is white.

In the next series of experiments methods converting different length signals to the same length signals (interpolation vs. zero padding) and proper signal representations (time vs. frequency) were evaluated. First, we visualize typical inputs to CNN for 5 LR and 5 HR subjects, i.e. zero padded signals expressed in time and frequency (magnitude spectrum) are shown in Fig. 7A,B, and time interpolated energy normalized signals expressed in time and frequency are in Fig. 7C,D.

Clearly, there are visible differences between LR and HR groups especially in the case of zero padded signals in the time domain (Fig. 7A) and in the frequency—magnitude spectra (Fig. 7B). In both cases that stems mainly from the differences in reading times (lengths of the recordings).

The overall results showing the performance of the suggested pre-processing methods, signal representations, and the tested CNN structures are listed in Table 1. For a better comparison results of other published methods are listed as well, using accuracy, true positive (TPR) and true negative (TNR) rates; the methods are listed in an accuracy-descending order. The highest accuracy (96.6 ± 2.9) was recorded in the case of magnitude spectrum of time interpolated signals. However, a magnitude spectrum representation was successful in both cases (zero padding and interpolation), e.g. the magnitude spectrum brought 0.7% and 23.1% improvements over the time domain representation for zero padded and interpolated signals, respectively. This may suggest it carries additional useful information to the reading times. The worst scoring candidate was obviously a time domain interpolated signal (73.5 ± 10.8) that is stripped off any reading time information and, moreover, is missing the benefits provided by a magnitude spectrum representation. When the CNN complexity is regarded the best results were achieved for a 3 layer CNN (4 layer CNN provides very similar results), that means such a small network is complex enough for a given processing, representation and dataset. Nevertheless when considering computational complexity of a CNN classifier compared to a SVM it is obvious that in general CNNs require more numerical operations than SVM. On the other hand, CNN performs all the major processing steps, e.g. detection, extraction, fusion of features and their classification at the same time. When the suggested CNN approach is used in the diagnosis, the screening time of the diagnosed person is incomparably longer than the classification itself, and thus it does not play an important role.

**Table 1:** Results for the tested signal pre-processing, representations, and CNN structures in comparison to other published methods.

Methods	Accuracy (%)	TNR (%)	TPR (%)
**Spectrum of time domain interpolated signal -3 layer CNN**	**96.6** ± **2.9**	95.4 ± 4.1	97.8 ± 2.1
**Spectrum of time domain interpolated signal -4 layer CNN**	96.3 ± 3.1	95.5 ± 3.9	97 ± 2.6
**Spectrum of time domain zero padded signal -3 layer CNN**	95.9 ± 3.4	–	–
**Spectrum of time domain interpolated signal-2 layer CNN**	95.6 ± 3.8	95.3 ± 4.3	96 ± 3.7
SVM-RFE^16^	95.6 ± 4.5	**95.7 ± 4.5**	95.5 ± 4.6
**Time domain zero padded signal-3 layer CNN**	95.2 ± 3.8	–	–
Hybrid Kernel SVM-PSO^5^	95.11	93.19	96.89
LR^5^	95.11	93.19	96.88
Hybrid Kernel SVM-PSO^21^	95 (94.5)^a^	89 (91.4)^a^	100 (97.2)^a^
SVM—Linear^5^	94.58	92.08	96.88
KNN^22^	94	92	93
KNN^5^	92.47	90.97	93.88
SVM-RBF^5^	92.44	86.52	**97.88**
SVM—Sigmoid^5^	92.44	86.52	**97.88**
RFC^5^	92.44	89.86	94.77
SVM-Linear^22^	93	92	94
SVM-RBF^22^	93	87	97
SVM-Sigmoid^22^	92	89	91
SVM -Hybrid^22^	93	88	90
RF^22^	91	94	91
Linear SVM^21^	90 (90.4)^a^	70 (87.8)^a^	94 (93.4)^a^
**Time domain interpolated signal-3 layer CNN**	73.5 ± 10.8	–	–

The suggested methods are in bold and all methods are sorted by their accuracies.

^a^We believe this is a correct value calculated as an average over tenfold results that were provided by the authors, but possibly incorrectly listed in their comparison table.

## Conclusions

We have designed, presented and analysed a novel holistic method for dyslexia detection in a text reading scenario. Unlike competing methods our system process entire signals with only minor pre-processing and two representations. This removes additional errors introduced by the feature detection-extraction methods. Furthermore, we let the machine learning represented by a CNN to learn implicit features and classify them at the same time. This is important as the usually extracted well-known features are not proven to be optimal for the detection process.

In comparison to the best scoring reference method SVM-RFE^16^ utilizing meticulously selected combination of global features we were able on average to improve the detection process from 95.6 to 96.6% by applying a holistic approach This was achieved by a rather simple signal pre-processing, representation (magnitude spectrum) and a suitable classification model (CNN).

Furthermore, our approach is between the 3 best scoring methods in terms of TPR, e.g. 97.8% compared to 97.88% as provided by SVM-RBR and SVM-linear methods. This can play a role in cases where TPR is more important than the overall performance. It is usually the case in medical diagnostics, where the misclassification of healthy patients would be less dangerous than not providing the treatment to seriously ill subjects. False negativity in this case can make it more difficult for a child to receive further education, as it will not be adequately adapted to his/her reading disorder^30^.

The reading time is predominant and provides by itself a high accuracy, i.e. 95.67%. This was also observed in other experiments i.e. the time domain zero padded signals (have implicit reading time information, i.e. number of padded zeros) reached a high accuracy of 95.6%, whereas for the time domain interpolated signals that are stripped off any time related information the detection accuracy dropped significantly to 73.5%.

As it was already mentioned, in the vast majority of cases, children with dyslexia read significantly slower than the healthy ones. However, there are still healthy children who naturally read slowly. The proposed method that uses the magnitude spectrum representation of time domain interpolated signals recorded the best accuracy, and it is obvious the time interpolation eliminates the effect of time. Thus this successful method is rather independent of the predominant reading time parameter that is observed in the dyslexia detection.

Another advantage of our system is that the input to a classifier is a vector of coordinates (x-axis) and thus there is no needed for other classification/detection of events that express different eye movement types and parameters e.g. number of fixations, length of fixations or length of saccades; moreover, there is no subsequent selection and combination of such features e.g. by recursive feature elimination. It should be noted that the detection of saccades and fixations is not errorless and may introduce additional noise that can affect the accuracy.

Observing the performance of our approach in relation to the complexity of CNN structures it is clear that relatively a simple structure (3–4 layers) can extract relevant information vital to classify dyslectic subjects in the text reading—eye tracking scenario.

The combination of eye-tracking and text reading task proves to be very powerful for the detection of dyslexia and other disorders. Thus it would be interesting to test if this or similar holistic approach would be as successful as it is in this task. For example, applying this approach for the detection of subjects with an increased risk of developing dementia based on eye-movements during a reading activity test^18^. Such test could complement some comprehensive assessment of mild cognitive impairment, such as Montreal Cognitive Assessment (MoCA)^31^.
